# Auxin‐mediated induction of *GAL* promoters by conditional degradation of Mig1p improves sesquiterpene production in *Saccharomyces cerevisiae* with engineered acetyl‐CoA synthesis

**DOI:** 10.1111/1751-7915.13880

**Published:** 2021-09-09

**Authors:** Irfan Farabi Hayat, Manuel Plan, Birgitta E. Ebert, Geoff Dumsday, Claudia E. Vickers, Bingyin Peng

**Affiliations:** ^1^ Australian Institute for Bioengineering and Nanotechnology (AIBN) the University of Queensland Brisbane Qld 4072 Australia; ^2^ School of Chemistry and Molecular Biosciences (SCMB) the University of Queensland Brisbane Qld 4072 Australia; ^3^ CSIRO Manufacturing Clayton VIC 3169 Australia; ^4^ CSIRO Future Science Platform in Synthetic Biology Commonwealth Scientific and Industrial Research Organisation (CSIRO) Black Mountain Canberra ACT 2601 Australia; ^5^ ARC Centre of Excellence in Synthetic Biology Queensland University of Technology Brisbane Qld 4000 Australia

## Abstract

The yeast *Saccharomyces cerevisiae* uses the pyruvate dehydrogenase‐bypass for acetyl‐CoA biosynthesis. This relatively inefficient pathway limits production potential for acetyl‐CoA‐derived biochemical due to carbon loss and the cost of two high‐energy phosphate bonds per molecule of acetyl‐CoA. Here, we attempted to improve acetyl‐CoA production efficiency by introducing heterologous acetylating aldehyde dehydrogenase and phosphoketolase pathways for acetyl‐CoA synthesis to enhance production of the sesquiterpene *trans*‐nerolidol. In addition, we introduced auxin‐mediated degradation of the glucose‐dependent repressor Mig1p to allow induced expression of *GAL* promoters on glucose so that production potential on glucose could be examined. The novel genes that we used to reconstruct the heterologous acetyl‐CoA pathways did not sufficiently complement the loss of endogenous acetyl‐CoA pathways, indicating that superior heterologous enzymes are necessary to establish fully functional synthetic acetyl‐CoA pathways and properly explore their potential for nerolidol synthesis. Notwithstanding this, nerolidol production was improved twofold to a titre of ˜ 900 mg l^−1^ in flask cultivation using a combination of heterologous acetyl‐CoA pathways and Mig1p degradation. Conditional Mig1p depletion is presented as a valuable strategy to improve the productivities in the strains engineered with *GAL* promoters‐controlled pathways when growing on glucose.

## Graphical abstract

We attempted to improve acetyl‐CoA production efficiency by introducing heterologous acetylating aldehyde dehydrogenase and phosphoketolase pathways for acetyl‐CoA synthesis to enhance production of the sesquiterpene nerolidol. Conditional Mig1p depletion is presented as a valuable strategy to improve the productivities in the strains engineered with *GAL* promoters‐controlled pathways when growing on glucose.

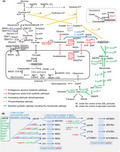

## Introduction

The commonly used yeast chassis organism *Saccharomyces cerevisiae* has been intensively engineered for improved production of various chemicals (Stephanopoulos, 2012; Jensen and Keasling, 2015; Nielsen, 2019). It has been shown to be a superior host for the synthesis of terpenoids (a.k.a. isoprenoids), a large group of natural products with a wide range of biological functions and commercial applications (Vickers *et al*., 2017; Alonso‐Gutierrez *et al*., 2018). For maximal terpenoid productivities (yield/titre/rate), it is crucial to optimize the metabolic networks, including sugar catabolic (central carbon) metabolism and terpene anabolism (Vickers *et al*., 2014; Meadows *et al*., 2016; Aslan *et al*., 2017; Vickers *et al*., 2017) as well as regulatory mechanisms to regulate these pathways (Stephanopoulos, 2012; Peng *et al*., 2015; Meadows *et al*., 2016; Shen *et al*., 2020).

**Fig. 1:**
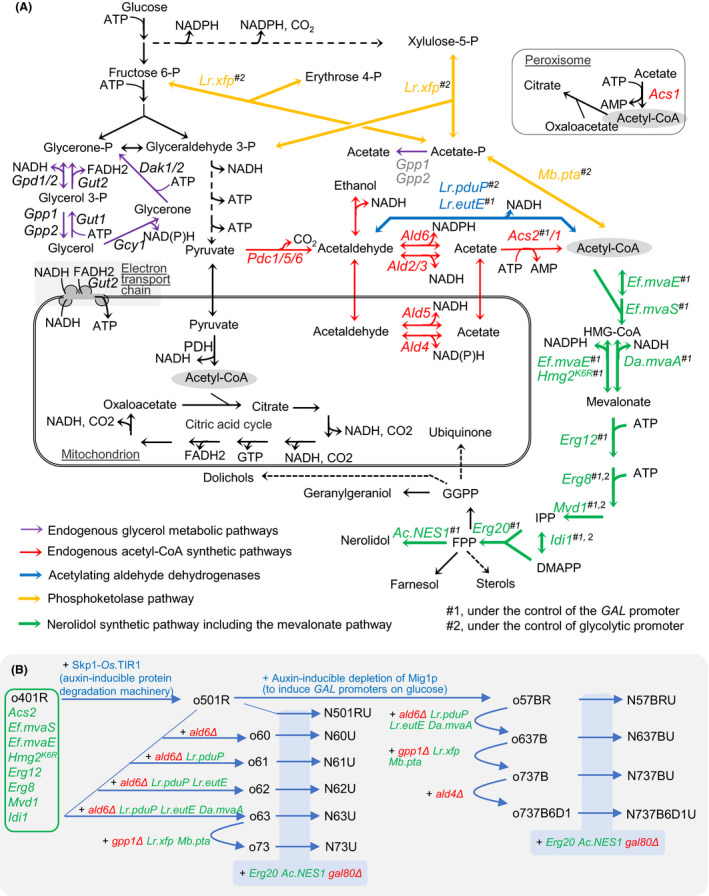
Metabolic pathways for the synthesis of acetyl‐CoA and nerolidol in *S. cerevisiae* (A) and diagram of strain construction processes (B). Acetyl‐CoA synthesis pathways: *Lr.xfp*, *Lactobacillus reuteri* fructose 6‐phosphate/xylulose 5‐phosphate phosphoketolase*; Mb.pta*, *Methanosarcina barkeri* phosphate acetyl‐transferase; *Lr.pduP* and *Lr.eutE*, *L. reuteri* acetylating aldehyde dehydrogenase; *Ald2/3/4/5/6*, ADA; and *Acs1/2*, acetyl‐CoA synthase. Terpene anabolic pathways: *Ef.mvaE*, *Enterococcus faecalis* acetoacetyl‐CoA thiolase/HMG‐CoA reductase; *Ef.mvaS*, *E. faecalis* HMG‐CoA synthase; *HMG2*, HMG‐CoA reductase 2; *Da.mvaA*, *Delftia acidovorans* NADH‐dependent HMG‐CoA reductase; *Erg12*, mevalonate kinase; *Erg8*, phosphomevalonate kinase; *Mvd1*, mevalonate pyrophosphate decarboxylase; *Idi1,* IPP:DMAPP isomerase, *Erg20*, farnesyl pyrophosphate synthetase, *AcNES1 Actinidia chinensis* *trans*‐nerolidol synthase. Glycerol metabolic pathway: *Gpd1/2*, glycerol‐3‐phosphate dehydrogenase; *Gpp1/2*, glycerol‐3‐phosphate phosphatase; *Gut1*, glycerol kinase; *Gut2*, glycerol‐3‐phosphate dehydrogenase; *Gcy1*, glycerol dehydrogenase; *Dak1/2*, dihydroxyacetone kinase. Others: Skp1, component of the SCF ubiquitin ligase complex; Os.TIR1, *Oryza sativa* auxin receptor; Mig1, transcriptional repressor in response to glucose; HMG‐CoA, 3‐hydroxy‐3‐methylglutaryl‐CoA; IPP, isopentenyl pyrophosphate; DMAPP, dimethylallyl pyrophosphate; FPP, farnesyl pyrophosphate.

In *S. cerevisiae*, the universal terpene precursors, isopentenyl pyrophosphate (IPP) and dimethylallyl pyrophosphate (DMAPP), are synthesised through the mevalonate pathway (Fig. 1; Vickers *et al*., 2017). The flux through this pathway is tightly regulated at both transcriptional and post‐transcriptional levels. In wild type yeast, the pathway synthesises structural terpenoids including sterols, as well as ubiquinones and prenyl groups for protein prenylation (Peng *et al*., 2017a,2017b). To increase mevalonate pathway flux for high‐level production of heterologous terpenoids, the enzymes from the mevalonate pathway must be deregulated and overexpressed (Bian *et al*., 2017; Vickers *et al*., 2017). Redirection of carbon flux to heterologous terpenoid products is achieved by expression of the heterologous terpenoid pathway enzymes and downregulation of enzymes catalysing the synthesis of native terpenoids (Peng *et al*., 2017a,2017b; Peng *et al*., 2018a,2018b). This approach targets the terpenoid anabolic pathways and has enabled the production of more than 40 g l^−1^ sesquiterpene in yeast fed‐batch fermentation (Westfall *et al*., 2012), but maximal productivities have not been reached (Gruchattka *et al*., 2013; Meadows *et al*., 2016).

Production of IPP and DMAPP via the mevalonate requires an input of acetyl‐CoA from central carbon metabolism (Fig. 1). Acetyl‐CoA metabolism in *S. cerevisiae* is compartmentalised to the cytosol, mitochondria and peroxisome (Pronk *et al*., 1996; Boubekeur *et al*., 1999; Chen *et al*., 2012; Fig. 1A). The mevalonate pathway is fed by cytosolic acetyl‐CoA, which is synthesised through the so‐called pyruvate dehydrogenase (PDH) bypass (Boubekeur *et al*., 1999; Fig. 1). The PDH bypass comprises of three steps of reaction catalysed by the pyruvate decarboxylases Pdc1p, Pdc5 and Pdc6p, cytosolic acetaldehyde dehydrogenase Ald6p, Ald2p and Ald3p or mitochondrial acetaldehyde dehydrogenase Ald4p and Ald5p (Boubekeur *et al*., 1999; Bakker *et al*., 2001; Boubekeur *et al*., 2001) and nuclear/cytosolic acetyla‐CoA synthase Acs2p (Acetate‐CoA ligase) and the cytosolic/peroxisomal acetyl‐CoA synthase Acs1p (Chen *et al*., 2012). Use of the PDH bypass to synthesize acetyl‐CoA has two key drawbacks: (i) the loss of carbon via CO_2_ production in the pyruvate decarboxylation step and (ii) the cost of two high‐energy phosphate bonds for acetyl‐CoA ligation (Bogorad *et al*., 2013; Henard *et al*., 2015; Meadows *et al*., 2016). Reducing the flux through the PDH bypass and introducing alternative acetyl‐CoA synthetic reactions increase the theoretical carbon yield of terpenoid production in yeast and decrease the amount of oxygen required for energy production (Meadows *et al*., 2016).

Alternative acetyl‐CoA synthesis can be engineered with, for example, an NAD^+^‐preferring acetylating aldehyde dehydrogenase (ADA; Fig. 1A, *pduP* and *eutE*, Kozak *et al*., 2014a,2014b; Meadows *et al*., 2016), the phosphoketolase pathway (Fig. 1A, *xfp* and *pta*, Bogorad *et al*., 2013; Meadows *et al*., 2016), cytosolic pyruvate dehydrogenase complex (Kozak *et al*., 2014a,2014b), pyruvate‐formate lyase (Kozak *et al*., 2014a,2014b) and ATP citrate lyase (Yu *et al*., 2018). Combining the ADA and the phosphoketolase pathway, an NADH‐preferring 3‐hydroxy‐3‐methyl‐glutaryl‐coenzyme A (HMG‐CoA) reductase (Fig. 1A, *mvaA*) can be introduced to oxidize the NADH produced in glycolysis and by NAD^+^‐dependent ADA. This combination provides a maximum theoretical yield of terpene products from glucose through the mevalonate pathway (Ma *et al*., 2011; Meadows *et al*., 2016). In combination with the strategies strengthening terpene anabolic pathway, this led to the production of sesquiterpene farnesene at a titre exceeding 130 g l^−1^ in an industrial process (Meadows *et al*., 2016).

Previously, we augmented the mevalonate pathway for improved production of sesquiterpene *trans*‐nerolidol (Peng *et al*., 2017a,2017b). We showed that restricting the consumption of acetyl‐CoA for lipogenesis redirected carbon flux towards nerolidol production (Lu *et al*., 2021). However, we have not introduced heterologous acetyl‐CoA pathways for improved terpene production. Meanwhile, the genes for nerolidol anabolism were overexpressed under the regulation of *GAL* promoters, in parallel with disruption of the *GAL* transcription repressor gene *GAL80* to enable diaxuie‐inducible expression from *GAL* promoters (Peng *et al*., 2017a,2017b; Peng *et al*., 2018a,2018b; Fig. 1A). In *gal80Δ* background strain, *GAL* promoters are repressed by glucose via Mig1p‐mediated repression (Frolova *et al*., 1999). This is problematic for characterisation of the effects of engineered acetyl‐CoA pathways on nerolidol production when glucose is used as carbon source. This might restrict industrial production in glucose‐pulse fed‐batch cultivation (Westfall *et al*., 2012). A regulatory mechanism is therefore required for conditional induction of *GAL* promoters on glucose.

In this study, we first introduced new heterologous acetyl‐CoA synthesis pathways in yeast with the aim to increase precursor availability for terpene production. To address the problem of a regulatory mechanism for conditional induction of *GAL* promoters on glucose, we implemented an auxin‐inducible degradation approach (Lu *et al*., 2021) to deliver auxin‐mediated degradation of the glucose‐dependent transcriptional repressor Mig1p. This provided high‐level expression of heterologous nerolidol pathway genes across the fermentation on different carbon sources (glucose and ethanol). Using this tool, we characterised the impact of engineered acetyl‐CoA synthetic pathways on nerolidol production and sugar metabolism.

## Results

### Engineered acetyl‐CoA synthetic pathways in nerolidol‐producing strains

We previously engineered *trans*‐nerolidol production in *S. cerevisiae* by overexpressing the mevalonate pathway and the genes encoding farnesyl pyrophosphate synthase and nerolidol synthase (terpene anabolic genes; Peng *et al*., 2017a,2017b; Peng *et al*., 2018a,2018b). The background mevalonate engineered pathway had one copy of *ACS2*, *Ef‐mvaE*, *Ef‐mvaS*, *HMG2^K6R^*, *ERG8*, *MVD1* and *IDI1* and two copies of *ERG12* overexpressed under the control of the *GAL* promoters; one copy of *ERG8*, *MVD1* and *IDI1* are overexpressed under the control of glucose‐inducible promoters (strain o401R). These modifications delivered titres of ˜ 400 mg l^−1^ nerolidol in flask cultivation (Peng *et al*., 2017a,2017b; Peng *et al*., 2018a,2018b). The auxin‐mediated protein degradation mechanism was previously engineered for investigation of novel metabolic engineering strategies in terpene‐overproducing strains (Lu *et al*., 2021). To apply this tool into the strains in this work, we introduced the fusion of *SKP1* (component of the SCF ubiquitin ligase complex) and *Oryza sativa* auxin receptor (*SKP1‐Os.TIR1*; Lu *et al*., 2021) into o401R to generate a new background strain o501R (Fig. 1B; Table 1; Table S3).

**Table 1:** *S. cerevisiae* strains used in this work.

Strain	Genotype	Resource/references
*S. cerevisiae*		
CEN.PK2‐1C	*MATa ura3‐52 trp1‐289 leu2‐3,112 his3Δ1*	Entian and Kötter (1998)
CEN.PK113‐5D	*MATa ura3‐52*	Entian and Kötter (1998)
ILHA series strains		
o401R	CEN.PK2‐1C derivative; *HMG2^K6R^(−152,‐1)::HIS3‐T_EFM1_<Ef.mvaS<P_GAL1_‐P_GAL10_>ACS2>T_ACS2_‐P_GAL2_> Ef.mvaE >T_EBS1_‐P_GAL7_* *pdc5(−31,94)::P_GAL2_> ERG12>T_NAT5_‐P_TEF2_>ERG8>T_IDP1_‐T_PRM9_<MVD1<P_ADH2_‐T_RPL15A_<IDI1<P_TEF1_‐TRP1* *ERG9(1336, 1336)::T_URA3_‐ P_GAL7_>MVD1>T_PRM9_‐P_GAL2_>ERG12>T_NAT5_‐T_IDP1_<ERG8<P_GAL10_‐P_GAL1_>IDI1>T_RPL15A_‐loxP‐ble‐loxP*	Peng *et al*. (2018a,2018b)
o501R	o401R derivative; *ERG9(1336, 1336)::T_URA3_‐ P_GAL7_>MVD1>T_PRM9_‐P_GAL2_>‐ERG12>T_NAT5_‐T_IDP1_<ERG8<P_GAL10_‐P_GAL1_>IDI1>T_RPL15A_‐P_ACS2_>SKP1‐Os.TIR1*	This work
o60	o501R derivative; *ald6(41, 1053)Δ*	This work
o61	o501R derivative; *ald6(41, 1053):: P_ADH1_* > *Lr‐pduP*> *T_PDC1_*	This work
o62	o501R derivative; *ald6(41, 1053):: P_ADH1_* > *Lr.pduP*> *T_PDC1_* > *P_Sb.GAL2_* >*Lr. EutE*	This work
o63	o501R derivative; *ald6(41, 1053):: P_Sk.GAL1_* >*Da.mvaA*>*T_PGK1_* > *P_ADH1_* > *Lr.pduP*> *T_PDC1_* > *P_GAL2_* >*Lr. EutE*	This work
o73	o63 derivative; *gpp1(79, 753)::P_Ag.TEF1_>hphMX6>T_Ag.TEF1_‐P_ENO2_> Lr.xfp>T_TPI1_‐P_TDH3_>Mb.pta>T_FBA1_*	This work
N501RU	o501R derivative; [pJT9R] *gal80*::*URA3*	This work
N60U	o60 derivative; [pJT9R] *gal80*::*URA3*	This work
N61U	o61 derivative; [pJT9R] *gal80*::*URA3*	This work
N62U	o62 derivative; [pJT9R] *gal80*::*URA3*	This work
N63U	o63 derivative; [pJT9R] *gal80*::*URA3*	This work
N73U	o73 derivative; [pJT9R] *gal80*::*URA3*	This work
oJ3	CEN.PK113‐5D derivative; *gal80::loxP‐kanMX4‐loxP*	Peng *et al*. (2017a,2017b)
o7B	oJ3 derivative; MIG1::CUP1‐AID*‐MIG1	This work
GB5J3	oJ3 derivative; *ura3(1, 704 )::KlURA3‐P_GAL1_‐yEGFP*	Peng *et al*. (2017a,2017b)
GJ38T	oJ3 derivative; *ura3(1, 704 )::KlURA3‐P_GAL1_‐yEGFP‐T_PGK1_‐P_ACS2_‐SKP1‐OsTIR1*	This work
G7B8T	o7B derivative; *ura3(1, 704 )::KlURA3‐P_GAL1_‐yEGFP‐T_PGK1_‐P_ACS2_‐SKP1 ‐OsTIR1*	This work
o57BR	o501R derivative; MIG1::CUP1‐AID*‐MIG1	This work
o637B	o57BR derivative; *ald6(41, 1053):: P_Sk.GAL1_* >*Da.mvaA*>*T_PGK1_* > *P_ADH1_* > *Lr.pduP*> *T_PDC1_* > *P_GAL2_* >*Lr. EutE*	This work
o737B	o637B derivative; *gpp1(79, 753)::P_ENO2_> Lr.xfp>T_TPI1_‐P_TDH3_>Mb.pta>T_FBA1_*	This work
N57BRU	o57BR derivative; [pJT9R] *gal80*::*URA3*	This work
N637BU	o637B derivative; [pJT9R] *gal80*::*URA3*	This work
N737BU	o737B derivative; [pJT9R] *gal80*::*URA3*	This work
N737B6D1U	o637B derivative; *gpp1(79, 753)::P_ENO2_> Lr.xfp>T_TPI1_‐P_TDH3_>Mb.pta>T_FBA1_* *ald4Δ* [pJT9R] *gal80*::*URA3*	This work

Symbol > or < indicates the direction of open reading frames.

From the new background strain o501R, five strains with a variety of acetyl‐CoA synthesis pathways and further genetic modifications were generated consecutively (Fig. 1B; Table 1). We first disrupted the major cytosolic acetaldehyde dehydrogenase encoding gene *ALD6* (strain o60) to reduce acetaldehyde conversion to acetyl‐CoA via the yeast’s inefficient native pathway. The *Lactobacillus reuteri* ADA gene *PduP* (*Lr*.*pduP*) was then expressed under the control of the *ADH1* promoter, resulting in strain o61. *PduP* expression creates an energy‐efficient short‐cut from acetaldehyde to acetyl‐CoA (Fig. 1). To augment the more efficient pathway, a second ADA encoding gene, *Lr*. *EutE,* was expressed under the control of the *Saccharomyces bayanus GAL2* promoter (Peng *et al*., 2018a,2018b; strain o62). Next, a codon‐optimised NADH‐preferring HMG‐CoA reductase gene from *Delftia acidovorans* (*Da.mvaA*) was introduced under the control of *Saccharomyces kudriavzevii GAL2* promoter, an alternative *GAL* promoter to avoid untargeted recombination (Peng *et al*., 2018a,2018b; strain o63). The aim of this step was to balance NADH metabolism by utilising the extra NADH produced by the introduced ADA enzymes, whilst concurrently augmenting mevalonate pathway flux. Finally, a hybrid phosphoketolase pathway, comprising an *L. reuteri* xylulose‐5‐phosphate/fructose‐6‐phosphate phosphoketolase gene (*Lr*.*xfp*) and a codon‐optimised *Methanosarcina barkeri* phosphate acetyltransferase gene (*Mb*.*pta*), was expressed under the control of glycolytic promoters, and *GPP1* was disrupted to reduce acetate formation from acetate‐phosphate (Bergman *et al*., 2016; Meadows*et al*., 2016), resulting in strain o73. In these background strains o501R (reference), o60 (*ald6Δ*), o61 (*ald6Δ*, *P_ADH1_‐Lr.pduP*), o62 (*ald6Δ*, *P_ADH1_‐Lr.pduP*, *P_Sb.GAL2_‐Lr.eutE*), o63 (*ald6Δ*, *P_ADH1_‐Lr.pduP*, *P_Sb.GAL2_‐Lr.eutE*, *P_Sk.GAL2_‐Da.mvaA*) and o73 (*ald6Δ*, *gpp1Δ*, *P_ADH1_‐Lr.pduP*, *P_Sb.GAL2_‐Lr.eutE*, *P_Sk.GAL2_‐Da.mvaA, P_ENO2_‐Lr.xfp, P_TDH3_‐Mb.pta*), the plasmid pJT9R carrying *GAL*‐promoters‐controlled nerolidol synthetic genes was introduced (Peng *et al*., 2017a,2017b). The transcriptional repressor gene *GAL80* was also disrupted in each strain to enable the diauxie‐coupled auto‐induction of the *GAL* promoters. These engineering steps resulted in the six nerolidol‐producing strains: N501RU (nerolidol reference strain), N60U, N61U, N62U, N63U and N73U respectively.

**Fig. 2:**
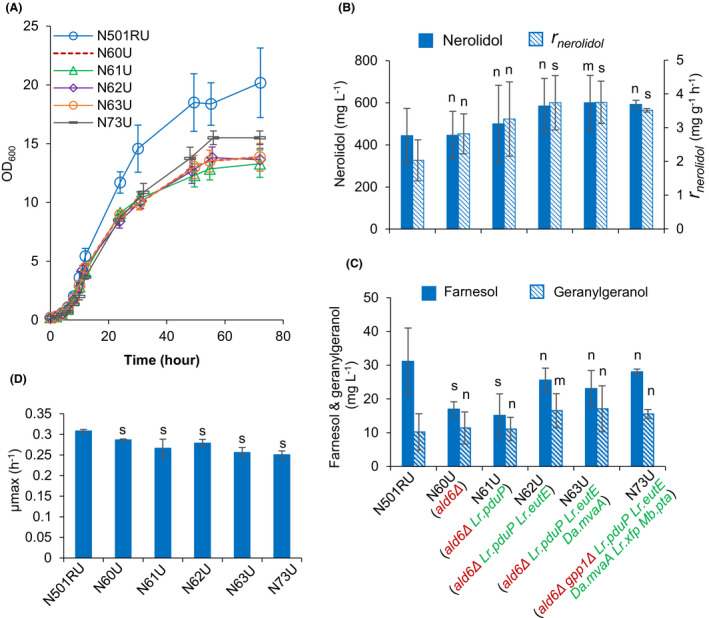
Characterising strains with engineered acetyl‐CoA synthesis pathways: (A) growth profiles; (B) nerolidol titre at 72 h and overall specific nerolidol production rate from 0 to 72 h; (C) farnesol and geranylgeraniol titre at 72 h; (D) maximum specific growth rate. Two‐phase flask cultivation on 20 g l^−1^ glucose was employed. For B, D and E, two‐tailed Welch’s t‐test was used for statistical analysis relative to N501RU: n, *P*> 0.1; m, *P* ∈ [0.05, 0.1]; s, *P* < 0.05. Mean values ± standard deviations are shown (*N* ≥ 4).

Growth and nerolidol synthesis in the five strains with modified acetyl‐CoA synthesis pathways and the reference strain N501RU were evaluated in flask cultivations in synthetic mineral medium (YNB) with 20 g l^−1^ glucose as the sole carbon source. The five acetyl‐CoA pathway engineered strains all had decreased final biomass production (Fig. 2A; *P* < 0.05) and slightly decreased specific maximum growth rates (Fig. 2D; *P* < 0.05) compared with the reference strain.

The nerolidol titres at 72 h for strains harbouring heterologous acetyl‐CoA synthesis pathways were slightly increased compared with the reference strains (Fig. 2B); however, those differences were not statistically significant (*P* > 0.05). Despite this, the overall specific nerolidol production rates were improved significantly by ˜ 70% in strain N73U (ADA pathway + NADH‐preferring HMG‐CoA reductase + PKA pathway), and ˜80% in the strains N62U (ADA pathway) and N63U (ADA pathway + NADH‐preferring HMG‐CoA reductase).

All strains produced small amounts of farnesol and geranylgeraniol (Fig. 2C), which are produced in the presence of excess prenyl phosphate accumulation by the action of non‐specific dephosphorylases (Peng *et al*., 2017a,2017b). While the farnesol titres in the strain N62U, N63U and N73U were similar to that in the reference, strains N60U and N61U showed an ˜ 50% decrease. Geranylgeraniol titres remained unchanged among all tested strains. Non‐specific prenyl alcohol production levels do not map to the nerolidol synthase‐driven specific production of nerolidol across strains, and it is unclear if these data are biologically relevant with the overall flux through the mevalonate pathway.

### Auxin‐mediated Mig1p depletion de‐repressing the *GAL* promoter on glucose

**Fig. 3:**
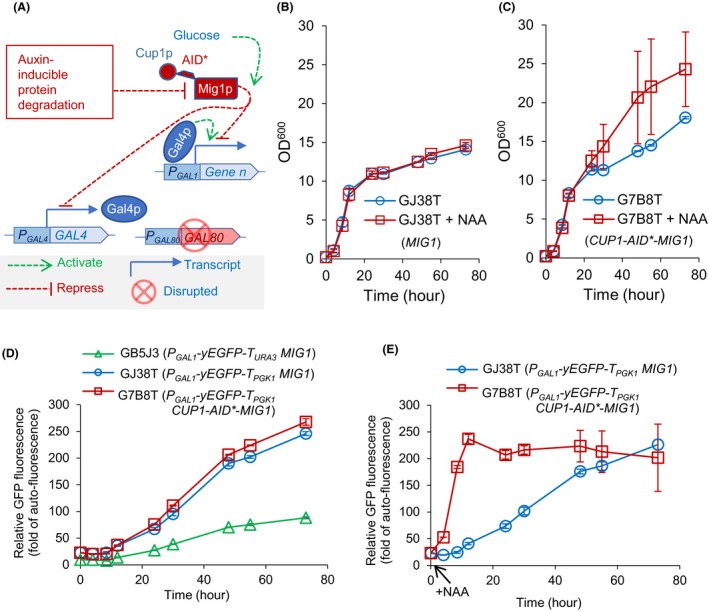
Engineering auxin‐mediated Mig1p depletion to induce the *GAL1* promoter. Yeast strains include GB5J3 (*gal80Δ MIG1 P_GAL1_‐yEGFP‐T_URA3_*), GJ38T (*gal80Δ MIG1 P_GAL1_‐yEGFP‐T_PGK1_‐P_ACS1_‐SKP1‐OsTIR1‐T_URA3_*) and G7B8T (gal80Δ CUP1‐AID*‐MIG1 P_GAL1_‐yEGFP‐T_PGK1_‐P_ACS1_‐SKP1‐OsTIR1‐T_URA3_). A. Schematic representation of the regulation on the *GAL1* promoter. B, C. The growth profile of engineered strains with or without naphthaleneacetic acid (NAA) addition at 1 h. D, E. GFP fluorescence levels in engineered strains under the conditions with or without NAA addition. NAA was added at 1 h. Mean values ± absolute errors are shown (*N* = 2).

We used the strong inducible *GAL* promoters to control the expression of heterologous genes for nerolidol production (Fig. 1A). *GAL* promoters are regulated by the galactose‐derepressible transcriptional repressor Gal80p and the glucose‐mediated transcriptional repressor Mig1p (Fig. 3A; Nehlin *et al*., 1991). When *GAL80* is disrupted, *GAL* promoters are auto‐induced in aerobic cultivations in the absence of, or after depletion of, glucose. This diauxie‐inducible expression system has been used to increase the production of various isoprenoids in metabolically engineered yeast by avoiding the metabolic burden caused by high‐level gene expression during the exponential growth phase and strongly driving the production in the post‐exponential growth phase. However, the disadvantage of applying *GAL* promoters in *gal80Δ* strains is that it prohibits the characterisation of acetyl‐CoA pathway‐engineered strains on glucose.

Double disruption of *GAL80* and *MIG1* results in constitutive activation of *GAL* promoters even in the presence of glucose (Nehlin *et al*., 1991). However, this is problematic if the induction of *GAL* promoter‐controlled genes, like constitutively overexpressing terpene synthetic pathways (Peng *et al*., 2017a,2017b; Peng *et al*., 2018a,2018b), causes metabolic burden/imbalance and slowed growth rates during the glucose phase. To avoid this, we exploited the auxin‐inducible protein degradation mechanism from plants for a conditional depletion of Mig1p (see schematic in Fig. 3A). This protein degradation mechanism has previously been reconstructed in yeast (Morawska and Ulrich, 2013) and consists of two key components: the F‐box protein TIR1 (auxin receptor) and the auxin‐inducible protein degron (AID). The system is powerful but is subject to substantial ‘basal degradation’ in the absence of auxin (Li *et al*., 2019; Sathyan *et al*., 2019). Fusion of the endogenous Skp1 protein (an essential component of the ubiquitin ligase complex in the degradation pathway) to TIR1 decreases the basal degradation, decreasing the leakiness and improving the inducibility of the system (Lu *et al*., 2021).

To test the effect of auxin‐mediated depletion of Mig1p on the expression level of *GAL* promoters, a Skp1p‐TIR1 fusion (Kanke *et al*., 2011) was expressed under the control of the constitutive *ACS2* promoter. *Mig1p* was tagged with a mini‐AID tag (AID*; *Arabidopsis thaliana* IAA17 truncated to amino acids 71–114; Morawska and Ulrich, 2013) fused to the metallothionein protein Cup1p. Fusion of Cup1p to AID* also decreases basal degradation in the absence of auxin (Lu *et al*., 2021).

A proxy construct was included to examine *GAL* promoter activity in the presence of Mig1p engineering. This construct consisted of a *GAL1* promoter fused to a yeast enhanced green fluorescent protein (*yEGFP*), allowing GFP fluorescence to report on *GAL1* promoter activity in response to various perturbations. In our previous works, we used the *URA3* terminator to control yEGFP transcription termination (strain GB5J3; Peng *et al*., 2017a,2017b). In the new construct, the *PGK1* terminator (Loison *et al*., 1989) was fused to downstream of yEGFP gene for cloning *SKP1‐TIR1* expression cassette into a single plasmid (pJAIDB58T; Table 2 and Supplementary Table 2). With yEGFP fused to the *PGK1* terminator, GFP fluorescence was ˜threefold higher than that under the control of the *URA3* promoter (Fig. 3D). Terminators are known to have a significant effect on expression strength (Curran *et al*., 2013); in this case, the *PGK1* terminator provides a higher expression level than the *URA3* terminator.

**Table 2:** Plasmids used in this work.

Plasmid	Features	References
pRS424	*E. coli*/*S. cerevisiae* shuttle plasmid; 2 μ, *TRP1*	Christianson *et al*. (1992)
pRS425	*E. coli*/*S. cerevisiae* shuttle plasmid; 2 μ, *LEU2*	Christianson *et al*. (1992)
pJT9R	pRS425: *T_RPL3_<ERG20<P_GAL1_‐P_GAL2_>Ac.NES1>T_RPL41B_*	Peng *et al*. (2017a,2017b)
pIR3DH8	Plasmid used to disrupt *GAL80* with *URA3* marker	This work
pML104	*E. coli*/*S. cerevisiae* shuttle plasmid; 2 μ, *URA3, P_TDH3_‐CRSIPR/Cas9‐T_ADH1_*, *P_SNR52_‐guideRNA‐swaI‐T_SUP4_*	Laughery *et al*. (2015)
pJCble	pML104; *URA3* marker was replaced with *ble* marker (Goldstein and McCusker, 1999)	This work
pIALD2	pRS424: *ALD6* (−125, 40)‐ *P_ADH1_* > *Lr.pduP*> *T_PDC1_*‐ *P_Sb.GAL2_* >*Lr. EutE*> *ALD6* (1054, 1749)	This work
pIALD2S	pRS424*: ALD6* (−125, 40)> *P_ADH1_* > *Lr.pduP*> *T_PDC1_*‐*ALD6* (1054, 1749)	This work
pIALD2E	pRS424: *ALD*(−125, 40)‐ *ALD6*(1054, 1749)	This work
pIALD2HMGr	pRS424: *ALD6*(−125, 40)> *P_Sk.GAL1_* >*Da.mvaA*>*T_PGK1_*‐*P_ADH1_* > *Lr.pduP*> *T_PDC1_*‐*P_GAL2_* >*Lr. EutE‐ALD6*(1054, 1749)	This work
pIPKA2	pRS424: *GPP1*(−220,80)> *P_ENO2_* >*Lr.xfp*>*T_TPI1_*‐*P_TDH3_* >*Mb.pta*>*T_FBA1_*‐*GPP1(754, 1400)*	This work
pIPKAH	pRS424: *GPP1*(−220,80)> *P_Ag.TEF1_>hphMX6*(Goldstein and McCusker, 1999)*>T_Ag.TEF1_‐P_ENO2_> Lr.xfp>T_TPI1_‐P_TDH3_>Mb.pta>T_FBA1_*‐*GPP1(754, 1400)*	This work
pJT9R	pRS425: *T_RPL3_<ERG20<P_GAL1_‐P_GAL2_>Ac.NES1>T_RPL41B_*	Peng *et al*. (2017a,2017b)
pILGB5A	Yeast integration plasmid; *P_URA3_‐KlURA3‐T_AgTEF1_‐P_GAL2_‐yEGFP‐T_URA3_*	Peng *et al*. (2015)
pJAIDB58T	pILGB5A; *P_URA3_‐KlURA3‐T_AgTEF1_‐P_GAL2_‐yEGFP‐ T_PGK1_‐P_ACS2_‐SKP1 ‐OsTIR1‐T_URA3_*	This work

Symbol > or < indicates the direction of the open reading frame.

Further engineering steps delivered two strains. The control strain GJ38T had the *gal80* disruption, the *P_GAL1_‐yEGFP‐T_PGK1_* expression cassette and constitutive expression of *SKP1‐OsTIR1*. In addition to the modifications found in GJ38T, strain G7B8T had the chromosomal *MIG1* fused with the AID*‐CUP1 fusion. Comparison between these strains allowed specific examination of the effect of Mig1p degradation on *GAL* promoter activity.

Growth and GFP fluorescence of GJ38T (*MIG1*) and G7B8T (CUP1‐AID*‐MIG1) were examined in flask cultivations using MES‐buffered mineral medium with 20 g l^−1^ glucose as the carbon source (Fig. 3). Glucose is expected to be depleted after 12 h and then yeast started to use ethanol as the carbon source (Peng *et al*., 2015; Peng *et al*., 2017a,2017b). Both strains showed similar growth patterns during the exponential growth phase of the culture in the presence or absence of synthetic auxin analog NAA. However, the strain with the AID*‐tagged *MIG1* (G7B8T; Fig. 3C) grew faster and produced more biomass during the post‐exponential growth phase compared with the untagged strain (GJ38T; Fig. 3B). This occurred in both the presence of NAA (added at 1 h cultivation) and in the absence of NAA. The effect was more marked in the presence of NAA, although very high variability of growth rate was also observed in these strains. The increased growth in the absence of NAA is presumably due to residual leaky degradation of Mig1p (Lu *et al*., 2021). NAA had no effect in the absence of the AID* tag (strain expressing only *SKP1‐OsTIR1*; Fig. 3b). The increased biomass production in the presence of the Mig1p degradation system may be caused by lifting Mig1p‐mediated repression on respiratory metabolisms (Bonander *et al*., 2008; Fendt and Sauer, 2010).

Fluorescence patterns from the *GAL1* promoter reporter construct were very similar between the two strains in the absence of NAA, with low level fluorescence during the exponential (glucose) phase of the culture and a gradual increase after 12 h when glucose was depleted (Peng *et al*., 2015), over the remainder of the 72 h cultivation (Fig. 3D). Addition of NAA resulted in a dramatic change in fluorescence in the AID*‐tagged *MIG1* strain, with a sharp increase in fluorescence during the exponential (glucose) phase of the culture, peaking at 12 h at the same level that the other strains/conditions reached after 72 h, and remaining at this level over the rest of the culture (Fig. 3E). No effect on fluorescence was seen in the wild type *MIG1* strain expressing only the *SKP1‐OsTIR1* fusion. These data confirm effective modification of the *GAL1* promoter behaviour in the presence of an AID*‐tagged *MIG1*, presumably as the result of degradation of the Mig1 protein and consequent release of glucose‐mediated repression of the *GAL1* promoter.

### Auxin‐mediated depletion of Mig1p improves nerolidol production in acetyl‐CoA pathway‐engineered strains

Next, we tested the combined effect of *GAL* promoter induction on glucose and the synthetic acetyl‐CoA pathways for nerolidol production. We used strain o501R (mevalonate pathway augmented; auxin‐inducible protein degradation machinery gene *SKP1‐TIR1* expressed under the control of the *ACS2* promoter; Fig. 1B; Table 1), as a background strain. To introduce auxin‐mediated induction of *GAL* promoters into strain o501R, Cup1p‐AID* was tagged to the N‐terminus of Mig1p (strain o57BR). Three nerolidol‐producing strains were built from strain o57BR: N57BRU, with auxin‐mediated degradation of Mig1p and nerolidol production (reference strain); N637BU, with auxin‐mediated degradation of Mig1p, an ADA pathway (*ald6Δ*, *P_ADH1_‐Lr.pduP*, *P_Sb.GAL2_‐Lr. EutE*) and nerolidol production; and N737BU, with auxin‐mediated degradation of Mig1p, an ADA pathway and a phosphoketolase pathway (*ald6Δ*, *gpp1Δ*, *P_ADH1_‐Lr.pduP*, *P_Sb.GAL2_‐Lr. EutE*, *P_ENO2_‐Lr.xfp* and *P_TDH3_‐Mb.pta*), and nerolidol production. These three strains were characterised under the induction of the *GAL* promoter controlled genes by adding NAA during the pre‐cultivation and again to the main culture immediately after inoculation.

**Fig. 4:**
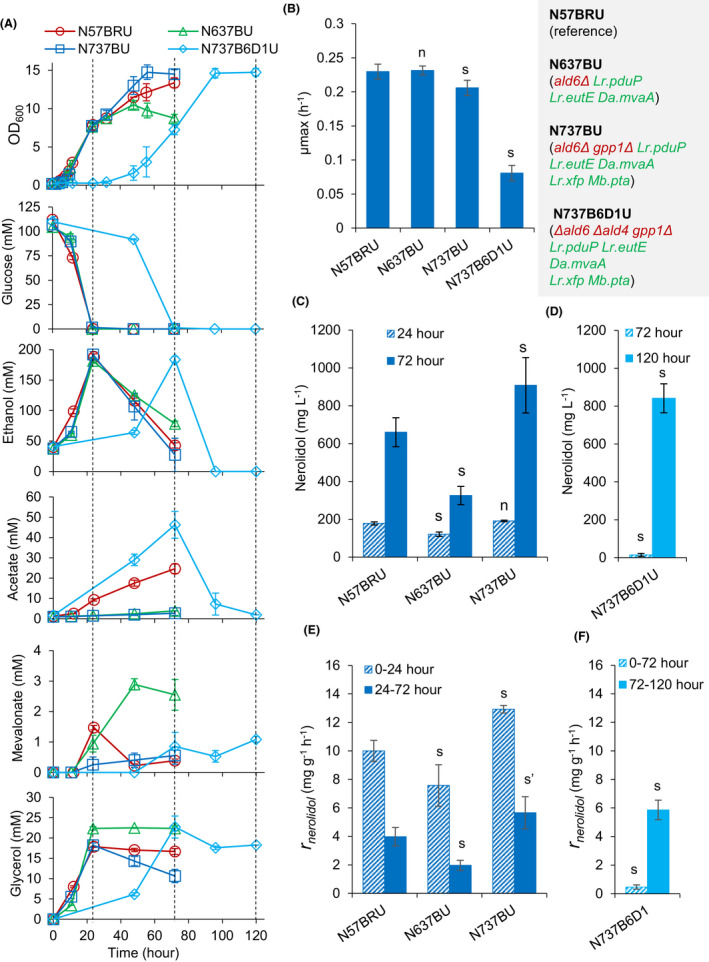
Characterising the strains with engineered acetyl‐CoA synthesis pathways and auxin‐mediated induction of *GAL* promoters: (A) growth (*N* = 4) and metabolic profiles (*N* = 2); (B) maximum specific growth rate (*N* = 4); (C and D), nerolidol titre (*N* = 4); (E and F), specific nerolidol production rate (*N* = 4). Two‐phase flask cultivation on 20 g l^−1^ glucose was employed, and NAA dissolved in ethanol was added in preculture and at the beginning of the cultivation. Dashed lines in A indicate 24, 72 and 120 h. For C–F, two‐tailed Welch’s t‐test was used for statistical analysis relative to N57BRU: n, *P* > 0.1; m, *P* ∈ [.05, 0.1]; s and s′ (calculated from log‐transformed data), *P* < 0.05. Mean values ± standard deviations are shown.

Compared with the strains with wild‐type Mig1p (Fig. 2A and D; N501RU, N63U, N73U), the Mig1p depletion strains N57BRU, N637BU and N737BU all showed decreased exponential growth in the presence of NAA (Fig. 4A and B). Strain N57BRU had a µ_max_ of ˜ 0.23 h^−1^ (Fig. 4B; with NAA addition) compared with its reference N501RU, which had a of µ_max_ ˜ 0.31 h^−1^ (Fig. 2D). Strain N57BRU also accumulated less biomass than N501RU during the post‐exponential phase (Figs 2A and 4A). These support the idea that Mig1p depletion or *GAL* promoter induction on glucose results in a metabolic burden or imbalance. Strain N637BU (ADA pathway + nerolidol production) showed growth arrest during the ethanol consumption phase (Fig. 4A), suggesting a significant extra metabolic burden of additional nerolidol production in the presence of acetyl‐CoA metabolism modifications in this strain. Consistent with the observation for strain N73U (ADA pathway and phosphoketolase pathway; *MIG1*‐wildtype; Fig. 2C and E), strain N737BU (ADA pathway and phosphoketolase pathway; Mig1p depleted + nerolidol production) showed slower exponential growth rate compared with the other strains (Fig. 4B), and superior post‐exponential growth (Fig. 4A). Despite the decreased growth rate relative to N73U, no negative effect on the biomass yield was observed for N737BU (Figs 2A and 4A).

Nerolidol production in the strains N57BRU, N637BU and N737BU was evaluated by measuring the titres at 24 and 72 h and calculating the specific nerolidol production rates for the period from 0 to 24 h and from 24 to 72 h. These two periods approximately resemble the growth phases on glucose and ethanol respectively (Fig. 4A). At 24 h, i.e., on glucose, strain N57BRU produced ˜ 180 mg l^−1^ nerolidol (Fig. 4C). This is much higher than the 24 h titre (˜ 40 mg l^−1^) in a very similar strain previously engineered with *GAL*‐promoter‐controlled nerolidol synthetic pathway but in the absence of *MIG1* depletion (strain N391DA; Peng *et al*., 2017a). The final nerolidol titre of ˜ 660 mg l^−1^ at 72 h of N57BRU (Fig. 4C) was significantly higher than titres achieved with strain N391DA 392 mg l^−1^ (Peng *et al*., 2017a,2017b) and N501RA (444 mg l^−1^; Fig. 2B; two‐tailed Welch’s *t*‐test, *P* = 0.04), which are comparable to each other. Nerolidol production in N637BU (ADA pathway) was only slightly lower at 24 h than strain N57BRU (reference strain), but significantly lower at 72 h (about half; Fig. 4C). This is consistent with the growth arrest phenotype during the ethanol phase (Fig. 4A), which suggests a major metabolic perturbation in this strain. However, strain N737BU (ADA pathway + phosphoketolase pathway) accumulated ˜ 910 mg l^−1^ nerolidol at 72 h, an improvement of ˜40% compared with N57BRU (Fig. 4C). For all three strains, the specific nerolidol production rate on glucose was more than twofold higher than that on ethanol (Fig. 4E).

Other extracellular metabolites acetate, mevalonate and glycerol were also measured (Fig. 4A). Compared with the reference N57BRU, acetate production was reduced in the acetyl‐CoA engineered strains. Mevalonate was secreted by all three strains (Fig. 4A), indicating an imbalance of pathway enzyme levels or an energy limitation of the ATP‐dependent downstream steps. In strain N57BRU, mevalonate peaked at 24 h and was consumed in the following ethanol growth phase. In contrast, strain N637BU (ADA pathway) continued to produce mevalonate into the ethanol phase. Production peaked at 48 h (concomitant with growth arrest) and the mevalonate was not re‐assimilated. Strain N737BU (ADA pathway + phosphoketolase pathway) produced only minor amounts of mevalonate. All strains exhibited similarly high glycerol formation during the glucose phase (Fig. 4A). Consistent with the reported deficiency of the parental *S. cerevisiae* CEN.PK strain in glycerol utilisation (Swinnen *et al*., 2013; Ho *et al*., 2017), strains N57BRU and N637BU did not consume glycerol during the ethanol phase (Fig. 4A). Interestingly, strain N737BU re‐assimilated glycerol during the ethanol phase.

These showed that Mig1p depletion improved nerolidol production when glucose was used as carbon source, overall nerolidol production in the reference strain without heterologous acetyl‐CoA pathways and in the strain with both ADA pathway and phosphoketolase pathway. In the strain with ADA pathway only, Mig1p depletion caused negative effects on growth and nerolidol production. It was also shown that engineered acetyl‐CoA pathways improved nerolidol production and causes perturbations on carbohydrate metabolism.

### Disruption of *ALD4* and *ACS2* in strains with engineered acetyl‐CoA synthetic pathways

To further force carbon flux through the engineered acetyl‐CoA synthetic pathways, we disrupted additional endogenous genes involved in acetyl‐CoA synthesis. We targeted *ALD4*, which encodes a mitochondrial ADA, and *ACS2*, which encodes the major acetyl‐CoA synthase (Fig. 1). *ALD4* and *ACS2* were disrupted separately in the background strains o60 (*ald6Δ*), o61 (*ald6Δ*, *P_ADH1_‐Lr.pdu*) and o73 (*ald6Δ*, *P_ADH1_‐Lr.pduP*, *P_Sb.GAL2_‐Lr.eutE*, *P_Sk.GAL2_‐Da.mvaA, P_ENO2_‐Lr.xfp, P_TDH3_‐Mb.pta*). The heterologous acetyl‐CoA synthesis pathways in strains o61 (ADA) and o73 (ADA + phosphoketolase pathway) are theoretically able to complement the deficiency caused by double disruption of *ALD6* and *ALD4* or double disruption of *ALD6* and *ACS2*. However, in contrast to the respective o60 derivatives without complementary genes for acetyl‐CoA synthesis, the *ald4*Δ derivatives and the *acs2*Δ derivatives of o61 and o73 did not show a restored growth on the synthetic mineral salt agar with glucose as the carbon source (Fig. S1). Supplementing acetate restored the growth deficiency caused by double disruption of *ALD6* and *ALD4* (Fig. S1). Acetyl‐CoA synthesis through acetylating ADA (Kozak *et al*., 2014a,2014b; Kozak *et al*., 2016; Meadows *et al*., 2016) and the phosphoketolase pathway (Meadows *et al*., 2016) have previously been shown to complement growth defects caused by the removal of endogenous reactions essential for acetyl‐CoA synthesis and to improve sesquiterpene production. These presumably indicate the novel gene combinations used here did not provide sufficient acetyl‐CoA in the presence of the disrupted endogenous acetyl‐CoA synthesis genes (*ald4Δ ald6Δ*; or *ald6Δ acs2Δ*).

Despite the apparent insufficiency in heterologous acetyl‐coA synthesis, we were interested in the impact of *ALD4* disruption on nerolidol production to confirm the phenotype for nerolidol production in the strain with major acetaldehyde dehydrogenases disrupted. We deleted this gene in strain N737BU (ADA + phosphoketolase pathways) and characterised the resulting strain N737B6D1U in the presence of NAA for *GAL* promoter induction (Fig. 4). Consistent with that double disruption of *ALD4* and *ALD6* caused growth deficiency (Fig. S1), the *ald4*Δ mutant showed extremely slow growth on glucose, with a maximum specific growth rate of ˜ 0.08 h^−1^ (Fig. 4A and B). Glucose utilisation was similarly slow and was accompanied by high production of acetate and glycerol (Fig. 4A).

Only ˜ 14 mg l^−1^ nerolidol (Fig. 4D) and < 1 mM mevalonate (Fig. 4A) were detected after 72 h (extended glucose phase). After glucose depletion, all the ethanol and 85% of the accumulated acetate were consumed within 24 h (Fig. 4A). The ethanol consumption rate was much faster than that of strains N57BRU, N637BU and N737BU. Like strain N737BU, N737B6D1 U also re‐utilized glycerol after glucose depletion. Interestingly, the nerolidol titre at 120 h was ˜ 840 mg l^−1^, ˜ 28% higher than that in the reference strain N57BRU at 72 h (a comparable time relative to the physiological stage of the cultures) and similar to N737BU at 72 h. While the specific nerolidol production rate on glucose was very reduced, it was much higher than the reference strain N57BRU during the ethanol phase (Fig. 4E and F). These results indicate that the heterologous acetyl‐CoA pathways engineered in this work were not active enough for acetyl‐CoA production to support cell growth, despite the improved overall nerolidol production.

## Discussion

Heterologous acetyl‐CoA pathways have been reconstructed previously in *S. cerevisiae* and have improved terpenoid production (Meadows *et al*., 2016; Vickers *et al*., 2017; Zhang *et al*., 2017). In this work, heterologous genes encoding ADA and phosphoketolase pathways were introduced in sesquiterpene nerolidol‐producing yeasts, attempting to construct carbon/energy‐efficient acetyl‐CoA synthesis pathways to improve isoprenoid (nerolidol) production. In addition to the acetyl‐CoA pathway engineering, we deployed an auxin‐mediated Mig1p depletion mechanism for *GAL* promoter induction on glucose, which improved nerolidol production on the glucose growth phase and overall nerolidol production. Combination of Mig1p depletion and engineered acetyl‐CoA synthetic pathway further improved nerolidol production and allowed us observe metabolic perturbation in these strains.

Previously, expressing various ADA genes in an *ald2/3/4/5/6*Δ background strain resulted in a fast‐growing strain with a maximum specific growth rate of 0.27 h^−1^ (Kozak *et al*., 2014a,2014b). Similar results were observed by expressing the ADA (EutE) from *E. coli*, in an *acs2Δ* background strain (Meadows *et al*., 2016). Expressing *xfp* from *Leuconostoc mesenteroides* and *pta* from *Clostridium kluyveri* enabled growth of an *acs2Δ ald6Δ* background strain on glucose (Meadows *et al*., 2016). However, while we observed enhanced specific nerolidol production in the strains with engineered acetyl‐CoA synthetic pathways in this work, they still exhibited growth defects when endogenous acetyl‐CoA pathways were inactivated. As the genes we used were from different species than the previous studies, this result underlines the importance of identifying optimal heterologous genes for efficient engineering.

Notwithstanding the deleterious physiological effects, the engineered heterologous pathways did deliver some of the targeted metabolic perturbation effects that we were aiming to achieve – including improved nerolidol production. Consistent with previous studies (Remize *et al*., 2000; Medina *et al*., 2010), acetate production was reduced in the *ald6Δ* strain (Fig. 4A; N637BU). While ADA pathway expression in combination with *ALD6* disruption (strain Ν63U) improved nerolidol production, negative effects were seen in the Mig1p‐depleted strain N637BU, including decreased biomass accumulation on ethanol, increased mevalonate and glycerol accumulation, and decreased nerolidol productivities. Mevalonate accumulation may indicate the presence of increased pathway flux and production/consumption imbalance. Additional modifications, i.e., disrupting *GPP1* and expression of phosphoketolase pathway genes *Lr.xfp* and *Mb.pta* (strain N737BU), alleviated the negative effects seen in strain N637BU and delivered almost 1 g l^−1^ nerolidol. Various phosphoketolases, including ones from *Lactobacillus*, have been expressed in *S. cerevisiae* previously (Sonderegger *et al*., 2004; Bergman *et al*., 2016) and expressing phosphoketolase has been shown to increase respiratory capacity (Bergman *et al*., 2019). This may contribute to improved nerolidol production in the strain harbouring phosphoketolase pathway. Further deletion of *ALD4* in this strain caused acetate accumulation on glucose growth phase (Strain N737B6D1 U; Fig. 4). Acetate accumulation was not shown previously in an *ald4Δ ald6Δ* strain (Remize *et al*., 2000). We presume that the presence of the engineered heterologous acetyl‐CoA synthetic pathways in this strain results in an imbalance which favours acetate production. However, more biochemical analysis would be required to understand these perturbation effects.

We applied auxin‐inducible protein degradation mechanism to investigate a new strategy allowing induction of *GAL* promoters on glucose. Previously, we showed that depletion of hexokinase 2 (Hxk2p), which interacts with Snf1p complex and coordinates Mig1p repression on *GAL* promoters (Ahuatzi *et al*., 2007), led to induction of *GAL* promoters on glucose and improved nerolidol production. Similarly, depleting Mig1p also delivered *GAL* promoter induction (Fig. 3) and improved nerolidol production (Fig. 4). This indicate that auxin‐inducible protein degradation is applicable to control depletion of a transcription factor for transcriptional regulation and that inactivating Mig1p can be adopted as a strategy to induce *GAL*‐promoter‐controlled pathway for improved production on glucose.

In summary, production of target sesquiterpene nerolidol was improved through engineering of acetyl‐CoA synthetic pathway and depletion of glucose‐dependent repressor Mig1p. The genes we introduced to construct ADA and phosphokinases pathways did not deliver enough acetyl‐CoA to support yeast growth but did cause improved production. It therefore is important to select optimal genes for construction of functional heterologous acetyl‐CoA pathways before applying them in metabolic engineering. This, on the other hand, shows that untargeted metabolic perturbation in metabolic engineering may cause improved production of target product. Depleting Mig1p was validated to induce *GAL* promoters and improve nerolidol production from engineered pathways under control of *GAL* promoters. This can be a general regulatory strategy for metabolic engineering in the yeast where *GAL* promoters are used to control pathway genes. However, instead of adding auxin in culture to trigger Mig1p depletion, dynamic mechanisms like quorum sensing (Lu *et al*., 2021) may be further exploited to control its depletion for application in industrial fed‐batch production.

## Experiment procedures

### Plasmid and strain construction

Strains and plasmids used in this study are listed in Tables 1 and 2. Primers and PCR products used in this study are listed in Table S1. Plasmid construction processes are described in Table S2. Strain construction processes are described in Fig. 1B and Table S3. Genes *Lr*.*pduP*, *Lr*.*eutE* and *Lr*.*xfp* were amplified from *Lactobacillus reuteri* genomic DNA, which was isolated from Blackmores Digestive Bio Balance^TM^ pills (purchased at Chemist Warehouse, Australia). The codon‐optimised gene sequences of *Delftia acidovorans mvaA* (Genbank: M24015.1), *Methanosarcina barkeri pta* (Genbank: AKB52167), *Oryza sativa TIR1* (Dharmasiri *et al*., 2005; Nishimura *et al*., 2009) and the minimal, auxin‐inducible degron AID* (Morawska and Ulrich, 2013), were synthesized by Integrated DNA Technologies (USA) as gBlocks® Gene Fragments.

### Two‐phase flask cultivation

Two‐phase flask cultivation was used to characterize the nerolidol‐producing strains (Peng *et al*., 2017a,2017b). Yeast cells were recovered from glycerol stocks by plating on yeast nitrogen base (YNB; with ammonia sulfate without amino acids) agar with 200 g l^−1^ glucose. YNB medium with 20 g l^−1^ glucose and 100 mM 2‐(N‐morpholino) ethanesulfonic acid (MES, Sigma‐Aldrich#M8250)‐ammonia buffer (pH 6) was used in preculture and flask cultivations. MES buffer was used to alleviate the alleviation of medium (Peng *et al*., 2015). Yeast cells were precultured to exponential phase in 15 ml MES‐buffered YNB‐glucose medium in a 50 ml Erlenmeyer flask (optical density at 600 nm from 0.8 to 5). Two‐phase flask cultivation was initiated by inoculating precultured cells to an OD600 of 0.2 in 23 ml MES‐buffered YNB‐glucose medium in a 250 ml Erlenmeyer flask; 2 ml dodecane were added to extract isoprenoid products. Flask cultivation was performed at 30°C and 200 rpm. A 500 mM ethanolic stock solution of the auxin analog NAA was prepared and added to the culture to a final concentration of 1 mM at indicated time points. For all cultivations, about 3 ml culture was sampled in the first 12 h for growth curve measurement. The dodecane phase and aqueous cell suspension were sampled for metabolite analysis and stored at −80°C. OD600 was measured using a SHIMAZU UV‐2450 UV–visible spectrophotometer or a WPA CO8000 cell density metre.

### Metabolite analysis

Nerolidol, farnesol and geranylgeraniol in dodecane samples were analysed using a reverse‐phase high‐performance liquid chromatography method (Peng *et al*., 2017a,2017b). If necessary, dodecane samples were diluted using dodecane to enable nerolidol concentration in the analytic range. Dodecane samples were diluted in a 40‐fold volume of ethanol. Ethanol‐diluted dodecane samples of 20 μl were injected into a Zorbax Extend C18 column (4.6 × 150 mm, 3.5 µm; Agilent, Santa Clara, CA, USA; part number: 763953‐902) with a guard column (SecurityGuard Gemini C18; Phenomenex, Lane Cove, NSW, Australia; part number: AJO‐7597). Analytes were eluted at 35°C at 0.9 ml min^−1^ using the mixture of solvent A (high purity water, 18.2 kΩ) and solvent B (45% acetonitrile, 45% methanol and 10% water), with a linear gradient of 5%–100% solvent B from 0 to 24 min, then 100% from 24 to 30 min and finally 5% from 30.1 to 35 min. Analytes of interest were monitored using a diode array detector (Agilent, Santa Clara, CA, USA; DAD SL, G1315C) at 202 nm wavelength. Analytical standards of *trans,trans*‐farnesol (96% purity; Sigma‐Aldrich, North Ryde, NSW, Australia; #277541), *trans*‐nerolidol (93.7% purity; Sigma‐Aldrich, North Ryde, NSW, Australia; #04610590) and geranylgeraniol (85% purity; Sigma‐Aldrich, North Ryde, NSW, Australia; #G3278), were used to prepare the standard curve for quantification.

### GFP fluorescence assay

GFP fluorescence was monitored in cells cultivated aerobically in 20 ml MES‐buffered YNB‐glucose medium in 100 ml flasks. NAA was added to the culture to a final concentration of 1 mM as indicated. Samples were taken at indicated time points; the GFP fluorescence in single cells was analysed immediately after sampling using a flow cytometer (BD Accuri™ C6; BD Biosciences, Franklin Lakes, NJ, USA). Cultures were diluted after 12 h with 10 volumes of water before flow cytometry analysis. GFP fluorescence was excited by a 488 nm laser and monitored through a 530/20 nm band‐pass filter (FL1.A); 10 000 events were counted per sample. The GFP fluorescence was expressed as percentage of the average background auto‐fluorescence from exponentially growing cells of the GFP‐negative reference strain GH4J3.

### Physiological feature calculation

The maximum specific growth rates (μ_max_) in flask cultivations were determined from the slope of the linear regression of the natural logarithm of the OD_600_ values versus time curve during the exponential growth phase. The biomass concentration was estimated using the OD600‐biomass correlation of 0.23 g l^−1^ of cell dry weight per unit of OD600 (Peng *et al*., 2012). The specific nerolidol production rate (*r_nerolidol_*, mg g^−1^ biomass h^−1^) was calculated by dividing the difference in the nerolidol titre (mg l^−1^) with the integral of biomass in defined time (g l^−1^ h^−1^).

## Funding Information

BP and this project were supported by a CSIRO Synthetic Biology Future Science Fellowship and the University of Queensland. IHF was supported by an Indonesia Endowment Fund for Education scholarship.

## Conflict of interests

The authors declare that they have no competing interests.

## Author contributions

BP and CEV conceived the study. IHF and BP performed experiments. MP performed the metabolite analyses. BP and IHF drafted the manuscript, and BEE, GD, and CEV revised the manuscript. All authors contributed to the result analysis and the discussion of the research. All authors read and approved the final manuscript.

## Supporting information


**Figure S1**. Testing growth complimentary effects of heterologous acetyl‐CoA synthetic genes in the strains with double disruption of *ALD6* and *ALD4* (A) or in the strains with double disruption of *ALD6* and *ACS2* (B). The strains in **a** are: strain o60 (*ald6Δ ALD4*), strain o60 with *ALD4* disruption (*ald6Δ*, *ald4Δ*), strain o61 with *ALD4* disruption (*ald6Δ*, *ald4Δ, Lr.pduP*), and strain o73 with *ALD4* disruption (*ald6Δ*, *ald4Δ, Lr.pduP, Lr.xfp, Mb.pta*); and were growth at 30 °C for 2 days. Strains in ^b^ are: strain o60 (*ald6Δ ACS2*), strain o60 with *ACS2* disruption (*ald6Δ*, *acs2Δ*), strain o61 with *ACS2* disruption (*ald6Δ*, *acs2Δ, Lr.pduP*), and strain o73 with *ACS2* disruption (*ald6Δ*, *acs2Δ, Lr.pduP, Lr.xfp, Mb.pta*); and were growth at 30 °C for 3 days. *ACS2* was disrupted by transforming PCR fragment #19 (Table S1).
**Table S1**. List of primers and PCR fragments used in this work. P_XXX_ and T_XXX_ indicate promoter and terminator sequence of gene XXX, respectively; red coloured sequences indicate a part that complementary to the DNA template; restriction enzyme sites used in cloning are shown in bold; 20‐mer CRISPR/Cas9 guide sequence was underlined in bold.
**Table S2**. Plasmid construction.
**Table S3**. Strain construction. DNA fragments refer to Supplementary Table S1, and plasmids refer to Table 2.
