# Supplementary material for: Auxin‐mediated induction of GAL promoters by conditional degradation of Mig1p improves sesquiterpene production in Saccharomyces cerevisiae with engineered acetyl‐CoA synthesis
Source: Microb Biotechnol. 2021 Sep 9;14(6):2627–42. doi: 10.1111/1751-7915.13880 (PMC8601163; doi:10.1111/1751-7915.13880)
Supplement: Supplementary file 1 — Figure S1. Testing growth complimentary effects of heterologous acetyl‐CoA synthetic genes in the strains with double disruption of ALD6 and ALD4 (A) or in the strains with double disruption of ALD6 and ACS2 (B). The strains in a are: strain o60 (ald6Δ ALD4), strain o60 with ALD4 disruption (ald6Δ, ald4Δ), strain o61 with ALD4 disruption (ald6Δ, ald4Δ, Lr.pduP), and strain o73 with ALD4 disruption (ald6Δ, ald4Δ, Lr.pduP, Lr.xfp, Mb.pta); and were growth at 30 °C for 2 days. Strains in b are: strain o60 (ald6Δ ACS2), strain o60 with ACS2 disruption (ald6Δ, acs2Δ), strain o61 with ACS2 disruption (ald6Δ, acs2Δ, Lr.pduP), and strain o73 with ACS2 disruption (ald6Δ, acs2Δ, Lr.pduP, Lr.xfp, Mb.pta); and were growth at 30 °C for 3 days. ACS2 was disrupted by transforming PCR fragment #19 (Table S1). Table S1. List of primers and PCR fragments used in this work. PXXX and TXXX indicate promoter and terminator sequence of gene XXX, respectively; red coloured sequences indicate a part that complementary to the DNA template; restriction enzyme sites used in cloning are shown in bold; 20‐mer CRISPR/Cas9 guide sequence was underlined in bold. Table S2. Plasmid construction. Table S3. Strain construction. DNA fragments refer to Supplementary Table S1, and plasmids refer to Table 2. [file MBT2-14-2627-s001.docx]

Supplementary information:

Auxin-mediated induction of GAL promoters by conditional degradation of Mig1p improves sesquiterpene production in *Saccharomyces cerevisiae* with engineered acetyl-CoA synthesis

Irfan Farabi Hayat^1, 2^, Manuel Plan^1^, Birgitta E. Ebert^1^, Geoff Dumsday^3^, Claudia E. Vickers^1,4,5^, Bingyin Peng^1, 4, 5*^,

1 Australian Institute for Bioengineering and Nanotechnology (AIBN), the University of Queensland, Brisbane, QLD 4072, Australia

2 School of Chemistry and Molecular Biosciences (SCMB), the University of Queensland, Brisbane, QLD 4072, Australia

3 CSIRO Manufacturing, Clayton, VIC 3169. Australia

4 CSIRO Future Science Platform in Synthetic Biology, Commonwealth Scientific and Industrial Research Organisation (CSIRO), Black Mountain, ACT 2601, Australia

5 ARC Centre of Excellence in Synthetic Biology, Queensland University of Technology, Brisbane, QLD 4001, Australia

* Corresponding author

Claudia E. Vickers (Claudia.vickers@csiro.au)

Bingyin Peng (bingyin.peng@qut.edu.au)

**Supplementary Table S1:** List of primers and PCR fragments used in this work. P_XXX_ and T_XXX_ indicate promoter and terminator sequence of gene XXX, respectively; red coloured sequences indicate a part that complementary to the DNA template; restriction enzyme sites used in cloning are shown in **bold**; 20-mer CRISPR/Cas9 guide sequence was **underlined in bold**.

| **No.** | **Overlap extension PCR fragment** | **PCR fragment** | **Primer name** | **Sequence (5’ 🡪 3’)** |
| --- | --- | --- | --- | --- |
| 1 | *ALD6(-125, 40)-P_ADH1_-Lr.pduP* | *ALD6(-125, 40)* from *S. cerevisiae* | PALDuparms | TTTTAACCAATAGGCCGAAATCGGCAAAATCCCTTA**ATTTAAAT***GTAATAAGAAGTTTGG* |
|  |  |  | PALDuparma | GAAAAAAATCGGAAAAGAGC**GGATCC***TCTTGACTGGTTCAGCAGTG* |
|  |  | *P_ADH1_* from *S. cerevisiae* | PALDADH1ps | CTGAACCAGTCAAGA**GGATCC***GCTCTTTTCCGATTTTTTTCTAAAC* |
|  |  |  | PALDADH1pa | TCGTTAATCTGCATTTT**GGTACC***TGTATATGAGATAGTTGATTGTATG* |
|  |  | *pduP* from *Lactobacillus reuteri* | PALDLrPduPs | CAACTATCTCATATACA**GGTACC**AAAATGCAGATTAACGATATTGAAAG |
|  |  |  | PALDLrPduPa | AATAATTAGAGATTAAATCGC*TTAGTACCAATTACGAACTGAG* |
| 2 | *T_PDC1_-P_GAL2_* | *T_DC1_* from *S. cerevisiae* | PALDPDC1ts | AGTTCGTAATTGGTACTAA*GCGATTTAATCTCTAATTATTAGTT* |
|  |  |  | PALDPDC1ta | TCTGGTGCGGTTGTGG**GCGGCCGC***TGTTCCTTAATCAAGGATACC* |
|  |  | *P_sbGAL2_* from *S. boulardii* | PALDSbGAL2ps | CCTTGATTAAGGAACA**GCGGCCGC***CCACAACCGCACCAGATCCG* |
|  |  |  | PALDSbGAL2pa | TATCATTAATCTGCATTTT**TCTAGA***TGCGAATGTGTGTATATTATATTAC* |
| 3 | *Lr.eutE-T_ald6_* | *eutE* from *Lactobacillus reuteri* | PALDEutwholes | CCG TGC CAA TGA ACT TGG TGT AGG |
|  |  |  | PALDEutwholea | GTGGTGGATCAGGGACAACATCAGAG |
|  |  |  | PALDEutEs | GTAATATAATATACACACATTCGCA**TCTAGA**AAA*ATGTTGATTAACGATATTGAAAGTGC* |
|  |  |  | PALDEutEa | AAGAAATGCAGGTTGGTACA**CTGCAG***TTAGTACCAATTACGAACTGAGAAC* |
|  |  | *T_ALD6_ (1054, 1749)* from *S. cerevisiae* | PALDdownarms | TTACGTAACTGGTATTAA***CTGCAG****TGTACCAACCTGCATTTCTTTC* |
|  |  |  | PALDdownarma | GTTCCTGGCCTTTTGCTGGCCTTTTGCTCA**ATTTAAAT***TCAATTCAATATGATT* |
| 4 | *P_SkGAL1_-Da.mvaA-T_PGK1_* | P_GAL1_ from *S. kudriavzevii* | pALDskGAL1psE | ATGACTAAGCTACACTTTGACACTGCTGAACCAGTCAAGA**GGATCC***ATGTTTTATTTGTAG* |
|  |  |  | pALDskGAL1ps | CTGAACCAGTCAAGA**GGATCC***ATGTTTTATTTGTAGAGTTCAATTTTTC* |
|  |  |  | pALDskGAL1pa | CAATCTGGAATCAGCAACCATTTT**AAGCTT***TATAGATTTTTATTTTTATCTTTTATGAG* |
|  |  | DamvaA from gBlocks^®^ | pALDHMGs | GATAAAAATAAAAATCTATA**AAGCTT**AAA*ATGGTTGCTGATTCCAGATTGCC* |
|  |  |  | pALDHMGa | ATCGATTTCAATTCAATTCAAT**CCCGGG***TTAAGCTCTTGCTCTTAATGC* |
|  |  | T_PGKI_ from *S. cerevisiae* | pALDPGKIts | GGCTGCATTAAGAGCAAGAGCTTAA**CCCGGG***ATTGAATTGAATTGAAATCGATAG* |
|  |  |  | pALDPGKIta | AGAAAAAAATCGGAAAAGAGC**GGATCC***TTCAAGCTTACACAACACGG* |
|  |  |  | pALDPGKItaE | CGAAATATTCCACGGTTTAGAAAAAAATCGGAAAAGAGC**GGATCC***TTCAAGCTTACAC* |
| 5 | *rhr2(-220,80)-P_ENO2_* | rhr2 (-220,80) from *S. cerevisiae* | RHR2UpArms | CAGTGAGCGCGCGTAATACGACTCACTATAGGGCGAATTG**ACTAGT***CTTTTTTCCTTTT* |
|  |  |  | RHR2UpArmsE | GGGCGAATTG**ACTAGT***CTTTTTTCCTTTTTTTTTCTTTTCTTACTCTG* |
|  |  |  | RHR2UpArma | GGCAAGAGTAGGATT**AGATCT***TGAGAGATGATGATGGTACCGTC* |
|  |  | P_ENO2_ from *S. cerevisiae* | ENO2ps | GACGGTACCATCATCATCTCTCA**AGATCT***AATCCTACTCTTGCCGTTGCC* |
|  |  |  | PPKENO2pa | GGAATCGTAATCAACAGCCATTTT**GGATCC***AAGTGGGCTTCAGAAAAAAGG* |
| 6 | *Lr.xfk* | LrxPK from *Lactobacillus reuteri* | PPKALrxPKs | CCTTTTTTCTGAAGCCCACTT**GGATCC**AAA*ATGGCTGTTGATTACGATTCC* |
|  |  |  | xPKa | AAGATAATATTTTTATATAATTATATTAATC**GCTAGC***TTACTTAAGACCCTTCCAAGTCC* |
| 7 | *T_TPI1_-P_TDH3_* | T_TPI1_ from *S. cerevisiae* | TPI1ts | TGGAAGGGTCTTAAGTAA**GCTAGC***GATTAATATAATTATATAAAAATATTATCTTCTT* |
|  |  |  | TPI1ta | GGTGACAAGGTCCTCGAAAATAG**GCGGCCGC***TGACGCCTCCAGTGAAAAAAC* |
|  |  | P_TDH3_ from *S. cerevisiae* | TDH3ps | GTTTTTTCACTGGAGGCGTCA**GCGGCCGC***CTATTTTCGAGGACCTTGTCACC* |
|  |  |  | PPKATDH3pa | GAAATCTTTTCCAAAAAAGTAACCATTTT**CTGCAG***TGAAGAAAACAATATTTTGGTGCTG* |
| 8 | *Mb.pta* | MbPTA from gBlocks^®^ | PPKAMbPTAs | CAGCACCAAAATATTGTTTTCTTCA**CTGCAG**AAA*ATGGTTACTTTTTTGGAAAAGATTTC* |
|  |  |  | PPKAMbPTAsada | ATGGTTACTTTTTTGGAAAAGATTTCTGAAAGAGCAAAGAAATTGAAC |
|  |  |  | PPKAMbPTAa | ATTAAAAAACTATATCAATTAATTTGAATTAAC**CTCGAG***TTACTTGTCCTGAGCAGCTGC* |
| 9 | *T_FBA1_-Trhr2* | T_FBA1_ from *S. cerevisiae* | FBA1ts | CAGCTGCTCAGGACAAGTAA**CTCGAG**GTTAATTCAAATTAATTGATATAGTTTT |
|  |  |  | FBA1ta | TAGTAGTTTTATCAAAAAAATAAAAGAAAA**TCTAGA**T*AAAACAGTTGTGAATAACAG* |
|  |  | Trhr2 from *S. cerevisiae* | RHR2downs | CTGTTATTCACAACTGTTTTA**TCTAGA***TTTTCTTTTATTTTTTTGATAAAACTACTA* |
|  |  |  | RHR2downa | CAAGCGCGCAATTAACCCTCACTAAAGGGAACAAAAGCTG**GAGCTC***AGGTGGTGG* |
|  |  |  | RHR2downaE | CACTAAAGGGAACAAAAGCTG**GAGCTC***AGGTGGTATCTTGCGTGCCC* |
| 10 | *hphMX6* | *hphMX6* from pAG32 | PxPKAhphs | GGTACCATCATCATCTCTCA*GAATACCCTCCTTGACAGTC* |
|  |  |  | PxPKAhpha | GCAACGGCAAGAGTAGGATTAGATCT*CGTTAGTATCGAATCGACAG* |
| 11 | *ALD6*-targeting guider sequence |  | PCRISPRs | TGAAAGTTGGTGCGCATGTTTCGGCGTTCGAAACTTCTCC*GCAGTGAAAGATAAATGATC* |
|  |  |  | PALDCRISPRALD6 | GCAGTGAAAGATAAATGATC **GTCAAGATCACACTTCCAAA**  GTTTTAGAGCTAGAAATAGC |
|  |  |  | PCRISPRa | ACTTTTTCAAGTTGATAACGGACTAGCCTTATTTTAACTT*GCTATTTCTAGCTCTAAAAC* |
| 12 | *GPP1*-targeting guider sequence |  | PCRISPRs | As above |
|  |  |  | PCRISPRERHR2 | GCAGTGAAAGATAAATGATC **CTCCAGAAAGCAGCAATGGC** GTTTTAGAGCTAGAAATAGC |
|  |  |  | PCRISPRa | As above |
| 13 | *MIG1*-targeting guider sequence |  | PCRISPRs | As above |
|  |  |  | PCRISPRMig1pN | GCAGTGAAAGATAAATGATC **CATTGGATATGGGCTTTGCA** GTTTTAGAGCTAGAAATAGC |
|  |  |  | PCRISPRa | As above |
| 14 | *CUP1-AID** donor | *CUP1* from S. cerevisiae | PCRISPRMig1pCUP1s | GAGAGTTGAGTATAGTGGAGACGACATACTACCATAGCC*ATGTTCAGCGAATTAATTAAC* |
|  |  |  | PADUCUP1a | CTTGGCTGGAGGTTTAGCTGGGTCTTTTGG*TTTCCCAGAGCAGCATGACT* |
|  |  | *AID** (gblock) | PADUAID*s | *CCAAAAGACCCAGCTAAACCTCCAGCCAAG* |
|  |  |  | PCRISPRMig1pAIDa | AACGTTAGACACTTGTGTCATTGGATATGGGCTTTGCAT*CTTAACAAAGGCAGCAGCTTC* |
| 15 | Verification primers |  | PCRISPRvMig1s | CATTTATTCTAGCTCGCTTGTAACTACAC |
|  |  |  | PCRISPRvMig1a | TCATGTGTCTCGTCTGATGTTCCAG |
|  |  |  |  |  |
| 16 | *ALD4*-targeting guide sequence |  | PCRISPRa | As above |
|  |  |  | PCRISPRs | As above |
|  |  |  | PdALD4CRISPR | GCAGTGAAAGATAAATGATC **CAATTGAAGTCTCCCAATGG** GTTTTAGAGCTAGAAATAGC |
|  |  |  | PCRISPRa | As above |
| 17 | *ALD4*-disrupting donor sequence |  | PdALD4RPAs | GAGATGGAAAATGAGGGGCGGGTGTAGGTAAGCAGAATGA*ATTGGGAGACTTCAATTGAG* |
|  |  |  | PdALD4RPAa | ATGACATATTTCTTCTTCCGTGGAAGGGTTAATG |
| 18 | Verification primer |  | PdALD4vs | ACTTCCGTCCACAGGTATCTTTTTCTCC |
| 19 | *KanMX4 to disrupt ACS2* |  | PdACS2s | ATACCTCTACCTTAAAATATATATATATATAGTATTCGAA *CAGCTGAAGCTTCGTACGC* |
|  |  |  | PdACS2a | AGCGAAATTTTATCTCATTACGAAATTTTTCTCATTTAAG *ATAGGCCACTAGTGGATCTG* |
| 20 | *ACS2*-disrupting donor sequence |  | PvdACS2s | CTGAATTTTAGCAACGATATCGCACC |
| 21 | Verification primer |  | PvACS3a | GTATTTTCGCTTTCCTAGCTGACCAG |
| 22 | *Partial_SUR7-P_URA3_-URA3-Partial_GAL80* | *Partial_SUR7* from *S. cerevisiae* | PGAL80uarms | GTAAAACGACGGCCAGTGAATTCGTTT*AAACGGCCGCCTCTGCCATG* |
|  |  |  | PGAL80uarma | ATCACTATCTCTTAGCATCT*TTAAACAGAGACATCGTCCG* |
|  |  | *P_URA3_-URA3* from *S. cerevisiae* | PGAL80URA3s | CGGACGATGTCTCTGTTTAA*AGATGCTAAGAGATAGTGAT* |
|  |  |  | PGAL80URA3a | CGAAGATCTCTTGTTGTAGTCCTCGAG*CTTTACAGTCCTGTCTTATTG* |
|  |  | *Partial_GAL80* from *S. cerevisiae* | PGAL80darms | CAATAAGACAGGACTGTAAAGCTCGAG*GACTACAACAAGAGATCTTCG* |
|  |  |  | PGAL80darma | GATTACGCCAAGCTTGCATGCGTTT*AAACCATTGCATTTATCCTGG* |
| 23 | *T_PGK1_-P_ACS2_-SKP1-Os.TIR1* | T_PGK1_ from *S.* cerevisiae | PAIDPGK1ts | GCTGAAGGTCGTCACTCCACCGGTGCTTAA*ATTGAATTGAATTGAAATCGATAG* |
|  |  |  | PAIDPGK1ta | TCTTGCAATCGCGCCAATTAATT*AAATAATATCCTTCTCGAAAGC* |
|  |  | *P_ACS2_* from *S. cerevisiae* | PAIDACS2ps | TCGAGAAGGATATTATTTAATTAATT*GGCGCGATTGCAAGAATTG* |
|  |  |  | PADACS2pa | CTTCTGGGAAATAGGTCATTTTGCTAGAA*TTTATTATTGTATTGATTTACTTTCCTG* |
|  |  | *SKP1* from *S. cerevisiae* | PAIDSKP1s | CAGGAAAGTAAATCAATACAATAATAAATTGCTAGCAAA*ATGGTGACTTCTAATGTTGTC* |
|  |  |  | PAIDSKP1adaa | TCTTCTTAAAGAAACAACCAGCACCCAAATCAAAGATACC*ACGGTCTTCAGCCCATTCAT* |
|  |  |  | PAIDSKP1adas | ATCTTTGATTTGGGTGCTGGTTGTTTCTTTAAGAAGAAGAGAAAAGTTGCTGGTGCTGGT |
|  |  |  | PAIDSKP1a | CTTCTTCTGGGAAATAGGTCATAGAAGTAAGGTC*ACCAGCACCAGCAACTTTTCTCTTCT* |
|  |  | *Os.TIR1* (gblock) | PPGE9TIR1s | CAAAAAACACATACATAAACTAAAAGCTAGCAAA*ATGACCTATTTCCCAGAAGAAG* |
|  |  |  | PPGE9TIR1a | CCTTCCCTTTGCAAATAGTCCT*TTACAAAATCTTTACGAAATTTGG* |
| 24 | *ERG9 terminator*-targeting guider sequence for removal of *loxP-ble-LoxP* | | PCRISPRa | As above |
|  |  |  | PCRISPRs | As above |
|  |  |  | PCRISPRd41 | GCAGTGAAAGATAAATGATC **CAGACTGCATAGGCCACTAG** GTTTTAGAGCTAGAAATAGC |
| 25 | *P_ACS2_-SKP1-TIR1* donor sequence for removal of *loxP-ble-LoxP* | | Pd41ACS2p | ATACCAATGCTAGCAGCTTACATAGGTGGTGGCACTACCA *ATTGCAAGAATTGATCTCCC* |
|  |  |  | Pd41TIR1a | TTCGGAGTTGTTTGTTTATGTTATTTGGCGCAGACT *TTACAAAATCTTTACGAAATTTGG* |
| 26 | Verification of *P_ACS2_-SKP1-TIR1* at *ERG9* terminator locus | | PvACS2p | GCCGATATTCGGTAGCCGATTCCGCTGG |
|  |  |  | PvERG9ta | GTTATCTTTAAGGATAGGCCTCTAC |
| 27 |  | *ble* from pUG66 (Gueldener, et al., 2002) | PPMLMarkers1 | GACTTAGATTGGTATATATACGCATATG *CAGCTGAAGCTTCGTACGCTG* |
|  |  |  | PPMLMarkera1 | ATTGATAATGATAAACTCGAACTGACTAGT *ATAGGCCACTAGTGGATCTG* |

**Supplementary Table S2**: Plasmid construction.

| Plasmid | Construction process |
| --- | --- |
| pIALD2 | *ALD6(-125, 40)-P_ADH1_-Lr.pduP, T_PDC1_-P_SbGAL2_, Lr.eutE-T_ALD6 (1054, 1749)_* fragments and *Sac1* digested pRS424 were co-transformed into *S. cerevisiae*. The assembled plasmid was recovered by transforming *E. coli* with total yeast genomic DNA. |
| pIALD2S | *Not1/Pst1* digested pIALD2 was treated with Mung Bean nuclease and ligated using Blunt/TA Ligase Master Mix from New England Biolabs (NEB) to generate pIALD2S |
| pIALD2E | *BamH1*/*Pst1* digested pIALD2 was treated with Mung Bean nuclease and ligated using Blunt/TA Ligase Master Mix from NEB to generate pIALD2E |
| pIALD2HMGr | *P_SkGAL1_-Da.mvaA-T_PGK1_* fragment and *BamH1* digested pIALD2 were assembled using NEBuilder® HiFi DNA Assembly Master Mix (NEB) to generate pIALD2HMGr. |
| pIPKA2 | *rhr2 (-220,80)-P_ENO2­_, Lr.xfk, T_TPI1_-P_TDH3_, Mb.pta, T_FBA1­_-T_rhr2_* fragments and *Kpn1*/*Sac1* digested pRS424 were co-transformed into the *S. cerevisiae*. The assembled plasmid was recovered by transforming *E. coli* with total yeast genomic DNA. |
| pIPKAH | *phpMX6* fragment and *BglII* digested pIPKA2 were assembled using NEBuilder® HiFi DNA Assembly Master Mix (NEB) to generate pIPKAH |
| pIR3DH8 | *Partial_SUR7-P_URA3_-URA3-Partial_GAL80* fragment and EcoRI/HindIII digested pUC19 were assembled using NEBuilder® HiFi DNA Assembly Master Mix (NEB) to generate pIR3DH8 |
| pJAIDB58T | *T_PGK1_-P_ACS2_-SKP1-Os.TIR1* and SpeI-digested pILGFPB5A were assembled using NEBuilder® HiFi DNA Assembly Master Mix (NEB) to generate pJAIDB58T |
| pJCble | Fragment ble (#27) and SpeI/NdeI-digested pML104 were assembled using NEBuilder® HiFi DNA Assembly Master Mix (NEB) to generate pJCble |

**Supplementary Table S3**: Strain construction. DNA fragments refer to Supplementary Table S1, and plasmids refer to Table 2.

| Strain | Construction process |
| --- | --- |
| o501R | Step 1: *ERG9 terminator*-targeting guider (#24), *P_ACS2_-SKP1-TIR1* donor (#25), and *Swa*I-digested pML104 was transformed into strain o401R.  Step 2: Episomal plasmid (*in vivo* assembled from pML104 and *ERG9* terminator-targeting guider) was removed from the resulted strain in Step 1 to generate strain o501R. |
| o60  o61  o62  o63 | Step 1: *ALD6*-targeting guider (#11), a donor DNA fragment (*Pme*I-digested pIALD2E, pIALD2S, pIALD2, and pIALD2HMGR, separately), and *Swa*I-digested pML104 was transformed into strain o501R.  Step 2: Episomal plasmid (*in vivo* assembled from pML104 and *ERG9* terminator-targeting guider) was removed from the resulted strain in Step 1 to generate strain o60, o61, o62, or o63, separately. |
| o73 | *Sac*I/*Spe*I-digested pIPKAH was transformed into o63 to generate o73. |
| N501RU  N60U  N61U  N62U  N63U  N73U | Step 1: Plasmid pJT9R was transformed into o501R, o60, o61, o62, o63, and o73, separately. More than ten clones were used in the following step.  Step 2: PmeI-digested pIR3DH8 was transformed into the resulted strains in Step 1, to generate N501RU, N60U, N61U, N62U, N63U, and N73U, separately. At least four verified clones were stored and characterised in the following characterisation. |
| o7B  o57BR | Step 1: *MIG1*-targeting guider sequence (#13), *CUP1-AID** donor (#14), and *Swa*I-digested pML104 was transformed into strain oJ3 and o501R, separately.  Step 2: Episomal plasmid (*in vivo* assembled from pML104 and *ERG9* terminator-targeting guider) was removed from the resulted strain in Step 1 to generate strain o7B and o57BR, separately. |
| GJ38T  G7B8T | *Swa*I-digested pJAIDB58T was transformed into oJ3 and o7B to generate GJ38T and G7B8T, separately. At least four verified clones were mixed and stored in a single glycerol stock. |
| o637B | Step 1: *ALD6*-targeting guider (#11), *Pme*I-digested pIALD2HMGR, and *Swa*I-digested pML104 was transformed into strain o501R.  Step 2: Episomal plasmid (*in vivo* assembled from pML104 and *ERG9* terminator-targeting guider) was removed from the resulted strain in Step 1 to generate strain o637B. |
| o737B | Step 1: *GPP1*-targeting guider (#12), *Sac*I/*Spe*I-digested pIPKA2, and *Swa*I-digested pML104 was transformed into strain o637B.  Step 2: Episomal plasmid (*in vivo* assembled from pML104 and *ERG9* terminator-targeting guider) was removed from the resulted strain in Step 1 to generate strain o737B. |
| N57BRU N637BU N737BU | Step 1: Plasmid pJT9R was transformed into o57BR, o637B, and o737B, separately. More than ten clones were used in the following step.  Step 2: *Pme*I-digested pIR3DH8 was transformed into the resulted strains in Step 1, to generate N57BRU, N637BU, and N737BU, separately. At least four verified clones were stored and characterised in the following characterisation. |
| N737B6D1U | Step 1: *ALD4*-targeting guider sequence (#16), *ALD4*-disrupting donor sequence (#17), and *Swa*I-digested pCRble.  Step 2: Plasmid pJT9R was transformed into the resulted strain in Step 1. More than ten clones were used in the following step.  Step 3: *Pme*I-digested pIR3DH8 was transformed into the resulted strains in Step 2, to generate N737B6D1U. At least four verified clones were stored and characterised in the following characterisation. |

**Supplementary Figure S1.** Testing growth complimentary effects of heterologous acetyl-CoA synthetic genes in the strains with double disruption of *ALD6* and *ALD4* (A) or in the strains with double disruption of *ALD6* and *ACS2* (B). The strains in **a** are: strain o60 (*ald6Δ ALD4*), strain o60 with *ALD4* disruption (*ald6Δ*, *ald4Δ*), strain o61 with *ALD4* disruption (*ald6Δ*, *ald4Δ, Lr.pduP*), and strain o73 with *ALD4* disruption (*ald6Δ*, *ald4Δ, Lr.pduP, Lr.xfp, Mb.pta*); and were growth at 30 °C for 2 days. Strains in ^b^ are: strain o60 (*ald6Δ ACS2*), strain o60 with *ACS2* disruption (*ald6Δ*, *acs2Δ*), strain o61 with *ACS2* disruption (*ald6Δ*, *acs2Δ, Lr.pduP*), and strain o73 with *ACS2* disruption (*ald6Δ*, *acs2Δ, Lr.pduP, Lr.xfp, Mb.pta*); and were growth at 30 °C for 3 days. *ACS2* was disrupted by transforming PCR fragment #19 (Supplementary Table S1).

**Reference**

Gueldener, U., Heinisch, J., Koehler, G., Voss, D., and Hegemann, J. (2002) A second set of loxP marker cassettes for Cre-mediated multiple gene knockouts in budding yeast, *Nucleic acids research* **30**: e23.
